# Calprotectin and N-telopeptide of Type I Collagen (NTx) as Gingival Crevicular Fluid (GCF) Biomarker in Peri-Implantitis Patients

**DOI:** 10.7759/cureus.28430

**Published:** 2022-08-26

**Authors:** Siddharth Swarup, Preeti Sabharwal, Manoj Kumar Meena, Anu Girdhar, Divya Ganjoo, Jatin Khippal

**Affiliations:** 1 Prosthodontics, Dr. D.Y. (Dnyandeo Yashwantrao) Patil Dental College and Hospital, Pune, IND; 2 Prosthodontics, Institute of Dental Studies and Technologies, Modinagar, IND; 3 Prosthodontics, Daswani Dental College and Research Centre, Kota, IND; 4 Prosthodontics, National Dental College & Hospital, Dera Bassi, IND; 5 Department of Periodontology, Shree Bankey Bihari Dental College, Ghaziabad, IND; 6 Department of Prosthodontics, Shree Bankey Bihari Dental College & Research Centre, New Delhi, IND

**Keywords:** ntx (composed of collagen type 1), gingival crevicular fluid (gcf), peri-implantitis, immune-inflammatory, calprotectin

## Abstract

Introduction: Formulation of various preventive and therapeutic strategies is possible only by a better understanding of the immune-inflammatory profile of peri-implant diseases. For understanding the changes and turnover of bone, various markers have been used in the past literature, out of which, N-telopeptide of Type I Collagen (NTx) is acknowledged to be the most reliable marker.

Aims and objectives: Assessment of calprotectin and NTx concentration in gingival crevicular fluid (GCF) around the implant sites in subjects suffering from peri-implantitis.

Materials and methods: In total, 70 healthy individuals were included in the present study. These patients had opted for dental implants within the last decade. After collecting the peri-implant crevicular fluid (PICF) and GCF, various examinations were carried out. PICF samples were obtained with the help of sterile paper available in the form of strips. The enzyme-linked immunosorbent assay (ELISA) technique was used for measuring the calprotectin and NTx. All the readings were obtained in nanograms per microliter of PICF. All the results were recorded and analyzed.

Results: The overall mean calprotectin and NTx values were observed to be in a significantly higher range within the sites suffering from peri-implantitis when compared with healthy locations. The calprotectin values and NTx levels were positively correlated with the mean values of periodontal parameters observed clinically.

Conclusion: Both calprotectin and NTx could be used as a biomarker signifying the presence of inflammation as well as bone resorption in patients suffering from peri-implantitis.

## Introduction

Dental implant procedures have become a common method of prosthetic rehabilitation of missing teeth nowadays. Peri-implantitis (PI) is frequently caused by a negative interaction between virulent microorganisms and dental implant-bearing tissues. It has been hypothesized that different individual-specific variables and characteristics decide the severity and extent of PI. The rate of peri-implant tissue breakdown and PI depends on several things, such as how well one takes care of one's mouth, etc., if one smokes, has systemic co-morbid conditions, and is overweight [1-3].

Formulating various preventive and therapeutic strategies is possible only by better understanding the immune-inflammatory profile of peri-implant diseases [4]. Various markers have been used in the past literature to understand the changes and turnover of bone, of which N-telopeptide of type I collagen (NTx) is acknowledged to be the most reliable marker. Osteoclastic activity is reflected by NTx as they do not degrade further and, therefore, represent the bone collagen degradation end-product. It has been proven in past studies that as a result of proteolysis of the collagen present within the bone by the activity of osteoclasts, immunologically reactive NTx is instigated [5]. Hence, the present study was planned to assess calprotectin and NTx levels in gingival crevicular fluid (GCF) around the implant sites in subjects suffering from PI.

## Materials and methods

**Figure 1:**
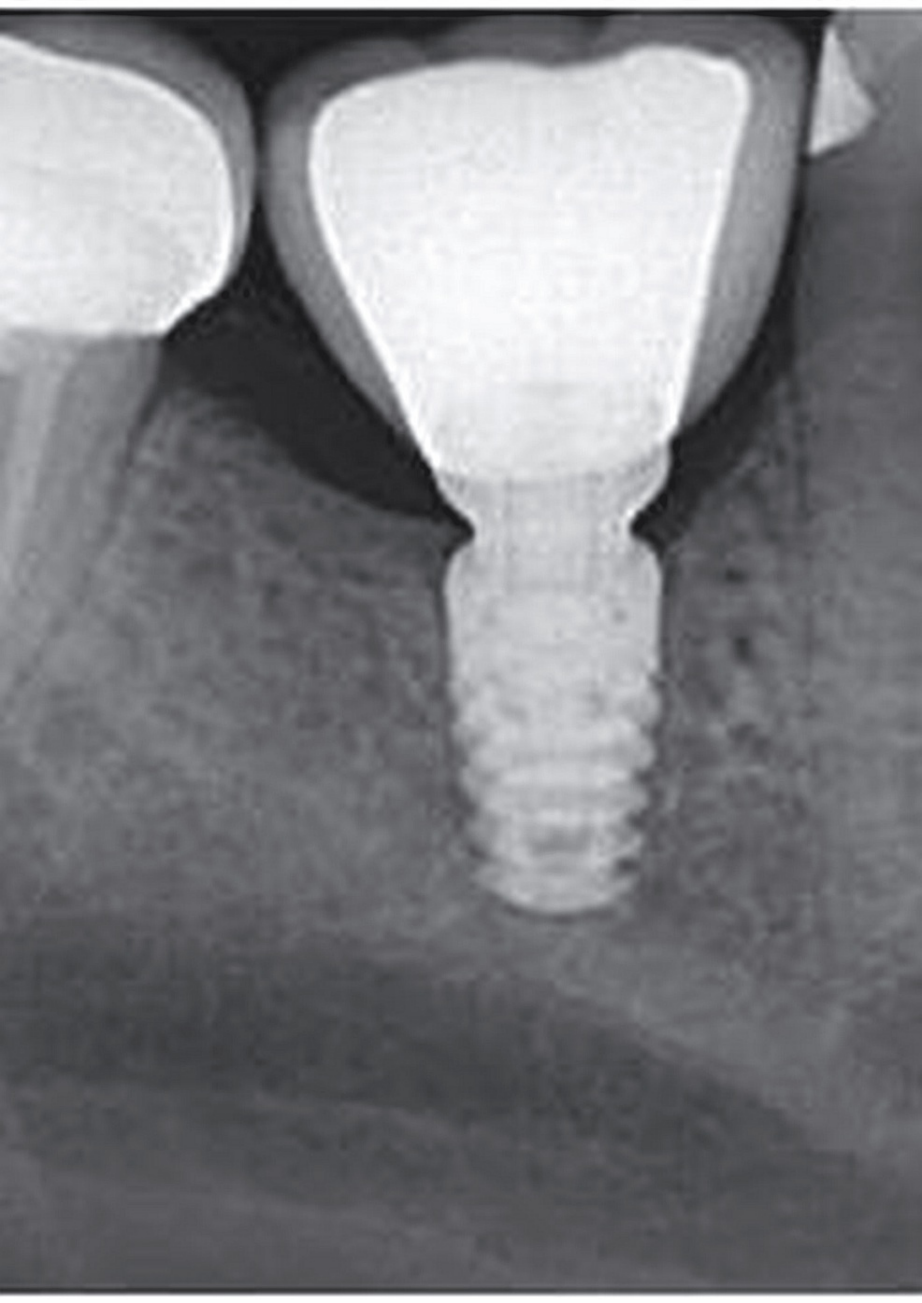
Radiographic image showing peri-implantitis

A total of 70 healthy individuals were included in the present study. These patients had opted for dental implants within the last decade. In this study, 35 peri-implant crevicular fluid (PICF) samples were collected from healthy peri-implant sites while 35 PICF samples were collected from diseased peri-implant sites which were having moderate PI. The current evaluative study was carried out within the periodontology department with ethical clearance from Dr. DY (Dnyandeo Yashwantrao) Patil Dental College and Hospital, Pune, India (PDCH/A56/2021). The aim of the study was to evaluate the calprotectin and NTx concentration in GCF around the implant sites in subjects with PI. Subjects were chosen for the study based on the following criteria: not having a systemic or metabolic disease, not taken any antibiotics in the past 90 days, and patients who got PI with a fair prognosis, were studied for an ailing implant, and were in good health after getting dental implants in the last 10 years. A radiographic image of the implant studied is shown in Figure 1.

**Figure 2:**
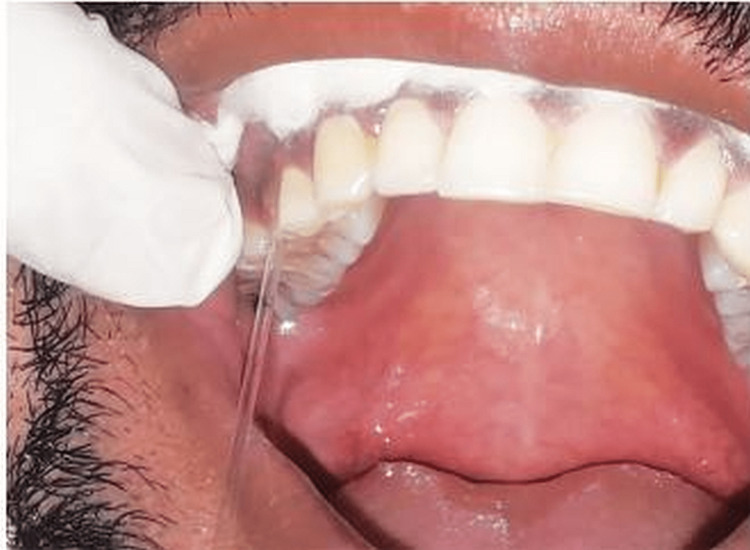
Collection of GCF sample GCF: gingival crevicular fluid

**Figure 3:**
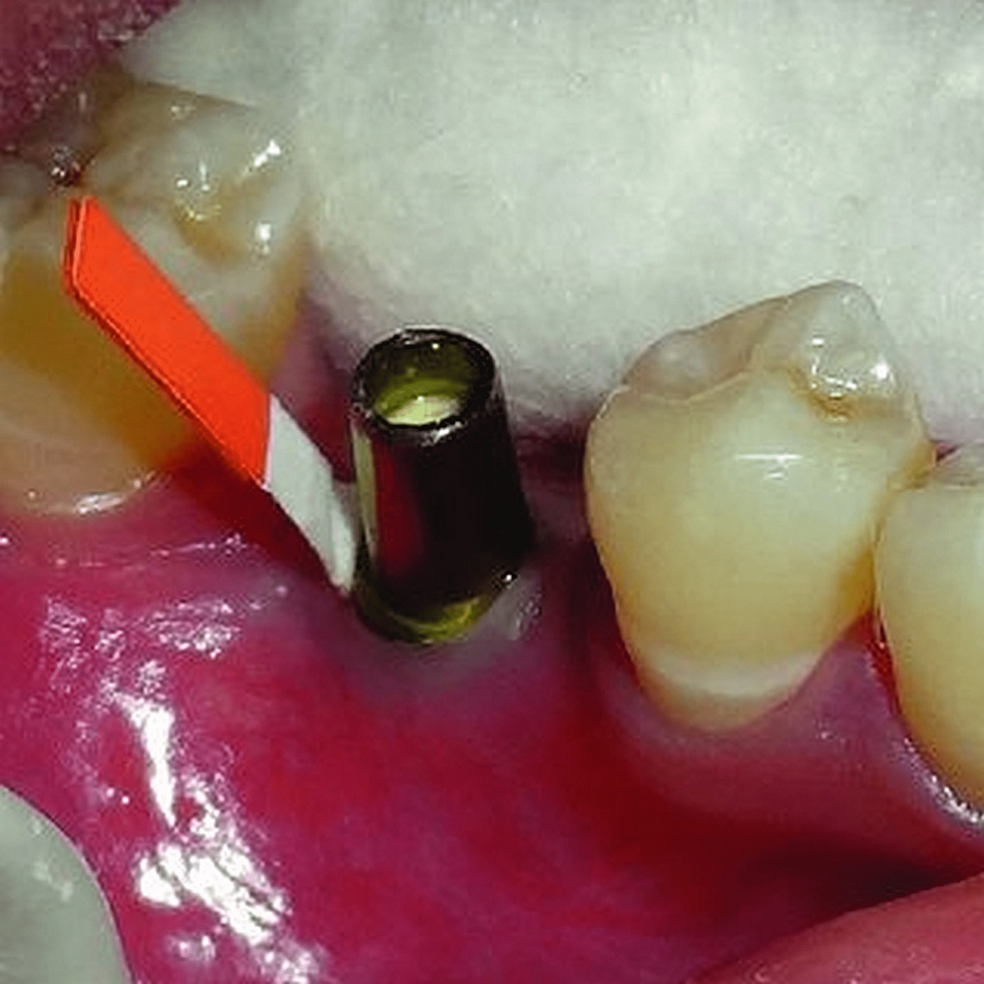
PICF collection from implant site PICF: peri-implant crevicular fluid

After collecting the PICF and GCF from the site where the deepest probing depth was observed in the same arch, various examinations were carried out. Samples of GCF and PICF were taken with the help of strips of sterile paper, as shown in Figure 2 and Figure 3.

All the readings were obtained in nanograms per microliter of PICF. All the results were recorded and analyzed. Gingival index (GI) values were calculated through the use of the modified Löe and Silness criteria [6]. Similarly, the modified version of the criteria postulated by Schei et al. was used to calculate the rate at which the alveolar bone displayed resorption [7]. PI was diagnosed at sites with a probing depth of more than 6 mm and GI values of 2 or more and evidence of bleeding from the free gingival sulcus on probing and associated bone loss.

As the same grade of PI was taken, it results in less bias in the study protocol as the stage of the disease can directly affect the biomarker level. PICF samples were obtained with the help of sterile paper available in the form of strips and appropriately prepared [8]. Cotton rolls were used for proper isolation of the sites from where the samples of PICF were collected. The next step was to remove all supra-gingival plaque and air dry the sites. The periopaper was then inserted into the prepared gingival crevices and held in place for 30 seconds. Thereafter, the quantity of PICF was evaluated with the help of a periotron. In the laboratory, the enzyme-linked immunosorbent assay (ELISA) technique was used for measuring calprotectin and NTx. Microsoft Excel (Microsoft Corporation, Redmond, Washington, United States) was used for recording the results. IBM SPSS Statistics for Windows, Version 20.0 (Released 2011; IBM Corp., Armonk, New York, United States) was used later to analyse the results. Significance levels were evaluated using the Chi-square test and the Mann-Whitney U test. All P-values less than 0.05 were considered to be significant.

## Results

In this study, 35 PICF samples were collected from healthy peri-implant sites based on the prevalence of the disease in that particular population while 35 PICF samples were collected from diseased peri-implant sites. The mean probing depth (after measuring all the sites: mesial, distal, buccal, and lingual) of 2.52 mm was seen in healthy sites, which was significantly lower than the mean probing depth of 4.96 mm seen in diseased sites (p-value 0.05). A mean GI score of 0 and 1.9 was obtained in the healthy and diseased peri-implant sites. Also, the mean bone loss rate among the healthy sites and diseased sites was found to be 23.5% and 44.1%, respectively. The results were found to be significant when the mean GI, as well as the mean bone loss rate, were compared among healthy and diseased sites (p-value 0.05). With reference to the sites that were healthy and diseased, the mean values of calprotectin within the PICF were found to be 176.1 and 43.3 ng/site, respectively (Table 1).

**Table 1:** Comparison of mean calprotectin and NTx levels µL: microlitre; PICF: peri-implant crevicular fluid; NTx: N-telopeptide of Type I collagen

Parameter	Diseased sites	Healthy sites	p-value
Mean calprotectin levels/site	176.1	43.3	0.001 (Significant)
Mean overall calprotectin concentration/µL of PICF	241.9	118.6	0.003 (Significant)
Mean NTx levels/site	7.96	2.92	0.001 (Significant)
Mean overall NTx concentration/µL of PICF	9.98	6.12	0.003 (Significant)

The overall mean concentration of calprotectin in the PICF among diseased and healthy sites was found to be 241.9 ng/L and 118.6 ng/L of PICF, respectively. The overall mean values of calprotectin were observed to be in a significantly higher range when a comparison was made between the diseased and healthy sites (p-value 0.05). Among the diseased and healthy sites, the mean NTx levels were found to be 7.96 ng/site and 2.92 gm/site, respectively. The overall mean values for the concentration of NTx in PICF were found to be 9.98 ng/L and 6.12 ng/L for both diseased and healthy sites, respectively.

The overall mean values of NTx calprotectin were observed to be in a significantly higher range in the diseased sites (p-value 0.05). During further evaluation, it was found that there was a positive relationship between the values of calprotectin and NTx in the PICF and the mean values of the clinical parameters.

## Discussion

The dental implant is one of the routine dental procedures performed globally. The relationship between teeth and implants used in dentistry has been found to be negatively correlated, particularly with regard to the CrossLaps levels as well as the b-glucuronidase performance in the GCF. However, a few previous studies have shown that the calprotectin values in the GCF are positively correlated with various periodontal parameters (both clinical and biochemical) in subjects suffering from periodontitis. Results from various experimental studies have shown that calprotectin has certain predictive properties that demonstrate clinical significance with regard to the development of gingival inflammation [9-11].

In this study, 35 PICF samples were collected from healthy peri-implant sites while 35 PICF samples were collected from diseased peri-implant sites. In total, the mean calprotectin values were observed in a significantly higher range in the diseased areas when compared with clinically healthy sites (p-value 0.05). Calprotectin is basically a protein associated with inflammation and is produced by various cells, both inflammatory and epithelial in nature. Elevation in levels of calprotectin has been observed in different inflammatory conditions, which include ulcerative colitis, rheumatoid arthritis, and cystic fibrosis. Early detection of calprotectin in the GCF has been reported in the literature [12,13]. Also, significantly higher GCF levels have been reported in patients with periodontal pathologies in comparison to healthy patients [14,15]. Also, results from previous studies indicate that periodontal disease activity can be predicted by the positive correlation obtained between GCF calprotectin levels and clinical periodontal indicators [16,17]. It is evident from these findings that calprotectin is a potent inflammatory biomarker to identify various diseased periodontal conditions. A relative scarcity of literature still exists when comparing calprotectin levels in GCF of clinically sound subjects and PICF of dental implant patients at disease sites [18]. NTx is produced as a by-product of cathepsin K-induced degradation of collagen (type 1) within the osteoclasts and is thereafter released both in urine as well as in blood. Hence, it can be considered an accurate biomarker to assess bone resorption [19-21].

In the current study, the overall mean NTx calprotectin was observed to be in a significantly higher range in the sites that were diseased when compared to the healthy ones (p-value 0.05). The levels of calprotectin and NTx within the PICF were observed to be positively correlated with the mean levels of various periodontal parameters observed clinically. In patients presenting with various osseous pathologies such as osteoporosis, bone cancers, and hyperparathyroidism, an elevation in the values of NTx, both in the urine as well as in the blood, has been observed, thereby signifying its role as a potential biomarker to identify various bone pathologies. However, no significant differences in relation to levels of NTx in GCF could be observed between the GCF of healthy sites and sites with periodontal pathologies [22-24]. There have been some contrasting reports as well, demonstrating the absence of NTx within the GCF in clinically healthy subjects, particularly after analyzing the levels of NTx in patients exhibiting periodontitis [23,24]. In the past, studies were conducted by Sakamoto and his associates to analyze and compare the levels of both calprotectin and NTx within the PICF around healthy and diseased implant sites. They analyzed a total of 35 subjects who underwent dental implant surgeries. Collection of the samples of the PICF was done from the peri-implant sites with the presence of periodontal pathologies (total number=40). Collection of the samples of the PICF was also done amongst healthy sites (total number = 34). Before collecting the GCF and PICF samples, they investigated the different clinical periodontal parameters. The levels of calprotectin and NTx were assessed using ELISA kits and, subsequently, a comparison was carried out between samples that were healthy and diseased. In order to predict peri-implant pathologies, an analysis of both calprotectin and NTx was performed using receiver operating characteristic (ROC) curve analysis. A significant correlation was observed between the levels of calprotectin within the PICF and the severity of periodontal pathologies. They came to the conclusion that the levels of calprotectin and NTx in the PICF of people with PI could be a sign of possible disease [25].

In 28 partially edentulous patients who were scheduled to undergo dental implant therapy (a total of 50 implants in all), Friedmann and his associates carried out a comparison of a new collagen-based membrane at the time of surgery scheduled at stage 2. At two- and three-year follow-up appointments, they measured clinical periodontal parameters like probing depth (PD), bleeding on probing (BoP), plaque index, GCF, and PICF volumes. Assessment of peri-apical radiographs was also done. An ELISA technique was used for assessing the levels of calprotectin as well as NTx within the GCF and PICF. They observed a significant elevation in the fluid volumes in both compartments. The significant rise corresponded with increasing NTx levels. However, the reduction in the total calprotectin levels in both the study groups was non-significant. The peri-apical radiographs [26] revealed a stable bone condition around dental implants.

Rapid immunochromatographic assays are used for rapid diagnostic methods for some diseases. The aim of this clinical study was to confirm what Kido et al. in a previous study assessed the quantity of calprotectin in PICF by employing immunochromatographic analysis. An assessment of 46 subjects who underwent dental implant procedures was carried out. PICD samples were obtained from the site showing signs of PI. They also evaluated the periodontal parameters, including PD, BOP, and GI. By employing immunochromatographic chips (IC) and the ELISA technique, calprotectin levels were assessed in the PICF samples. An IC reader was used to evaluate the calprotectin line’s density. After analysis of the results, they performed a ROC curve assessment to predict the extent of the inflammatory component in peri-implant pathology. They observed a significant correlation between the calprotectin IC reader value and its ELISA value. A significant correlation was also reported by them between calprotectin IC reader value and PD. They found that BOP-positive sites had much higher levels of calprotectin than BOP-negative sites [27].

NTx is formed as a result of the degradation product of collagen type I in osteoclasts. This is followed by their immediate release into the blood and urine. Hence, they can be a potential biomarker for bone resorption [28-30]. A few local risk variables are inherently variable in comparison to those present surrounding teeth, like the presence of metallic connections that cause infusion of metallic components because of tribocorrosion. This might explain why, though biomarkers are observed in periodontitis, their quantification significantly predicts PI [31-34]. Management for peri-implant pathologies is carefully chosen by Cumulative Interceptive Supportive Therapy (CIST). In these criteria, there is the employment of clinical periodontal variables for diagnosing peri-implant diseases. These variables alone can not be relied upon completely. Because of this, we need diagnostic and prognostic biomarkers like NTx as soon as possible to predict PI [35-38].

The limitations of this study include the modest sample size and the short duration of the study. This study provides evidence of the presence of calprotectin in diseased implant sites but gives little information on its aid in predicting possible disease in an otherwise healthy implant since the research provided indicates its absence in healthy implant sites altogether. More studies need to be conducted to assess its predictability in determining the prognosis of ailing implants.

## Conclusions

Peri-implant tissue has to be stable always and there are very few studies that are able to diagnose an ailing implant at an early stage. Hence, in light of the results of our study, it can be concluded that both calprotectin and NTx could be used as biomarkers to signify inflammation as well as bone resorption amongst patients with peri-implant pathologies. However, further studies are recommended for better exploration of the results. 
